# Gold Nanoparticle-Embedded Thiol-Functionalized Ti_3_C_2_T_x_ MXene for Sensitive Electrochemical Sensing of Ciprofloxacin

**DOI:** 10.3390/nano14201655

**Published:** 2024-10-15

**Authors:** Mari Elancheziyan, Manisha Singh, Keehoon Won

**Affiliations:** Department of Chemical and Biochemical Engineering, College of Engineering, Dongguk University-Seoul, 30 Pildong-ro 1-gil, Jung-gu, Seoul 04620, Republic of Korea; cheziyan@dongguk.edu (M.E.); manisha@dgu.ac.kr (M.S.)

**Keywords:** Ti_3_C_2_T_x_ MXene, gold nanoparticles, ciprofloxacin, portable electrochemical sensors, surface functionalization, antibiotics

## Abstract

The unregulated use of ciprofloxacin (CIPF) has led to increased resistance in patients and has threatened human health with issues such as digestive disorders, kidney disorders, and liver complications. In order to overcome these concerns, this work introduces a portable electrochemical sensor based on a disposable integrated screen-printed carbon electrode (SPCE) coated with gold nanoparticle-embedded thiol-functionalized Ti_3_C_2_T_x_ MXene (AuNPs-S-Ti_3_C_2_T_x_ MXene) for simple, rapid, precise, and sensitive quantification of CIPF in milk and water samples. The high surface area and electrical conductivity of AuNPs are maximized thanks to the strong interaction between AuNPs and SH-Ti_3_C_2_T_x_ MXene, which can prevent the aggregation of AuNPs and endow larger electroactive areas. Ti_3_C_2_T_x_ MXene was synthesized from Ti_3_AlC_2_ MAX phases, and its thiol functionalization was achieved using 3-mercaptopropyl trimethoxysilane. The prepared AuNPs-S-Ti_3_C_2_T_x_ MXene nanocomposite was characterized using FESEM, EDS, XRD, XPS, FTIR, and UV–visible spectroscopy. The electrochemical behavior of the nanocomposite was examined using CV, EIS, DPV, and LSV. The AuNPs-S-Ti_3_C_2_T_x_ MXene/SPCE showed higher electrochemical performances towards CIPF oxidation than a conventional AuNPs-Ti_3_C_2_T_x_ MXene/SPCE. Under the optimized DPV and LSV conditions, the developed nonenzymatic CIPF sensor displayed a wide range of detection concentrations from 0.50 to 143 μM (DPV) and from 0.99 to 206 μM (LSV) with low detection limits of 0.124 μM (DPV) and 0.171 μM (LSV), and high sensitivities of 0.0863 μA/μM (DPV) and 0.2182 μA/μM (LSV).

## 1. Introduction

The detection of ciprofloxacin (CIPF), a potent second-generation fluoroquinolone antibiotic, is pivotal across diverse domains due to its widespread usage against a broad range of bacterial infections [1]. Its mechanism of action is to inhibit bacterial DNA gyrase and topoisomerase IV enzymes, disrupting vital bacterial processes [2]. CIPF is prescribed for various infections like urinary tract infections, respiratory tract infections, skin and soft tissue infections, and gastrointestinal infections [3]. However, its responsible use is essential to mitigate antibiotic resistance and adverse side effects. CIPF finds extensive applications in animal husbandry, including cows, buffaloes, pigs, and other agricultural livestock. It serves multiple functions, such as disease management, prevention, and boosting growth rates, thereby enhancing overall production efficiency in these animals [4]. Using elevated levels or incorrect methods of administering CIPF in animal husbandry, particularly among dairy breeders, can lead to the accumulation of CIPF residues in milk. Consuming milk with CIPF residues can lead to detrimental effects on the body. Higher concentrations pose severe risks like nausea, tendonitis, central nervous system effects, and allergic reactions, besides increasing susceptibility to *Clostridium difficile* infection and organ damage [5]. Therefore, it is crucial for breeders to follow proper guidelines and dosages to minimize these risks and ensure the safety of dairy products. Detecting CIPF in water is also indispensable owing to its potential for both ecological and health ramifications [6]. CIPF contamination can disturb aquatic biodiversity and catalyze the proliferation of antibiotic-resistant strains. Compliance with stringent regulatory frameworks governing pharmaceutical residues in water is vital to public health and environmental integrity. Moreover, precise monitoring facilitates the identification of pollution sources, enabling targeted mitigation strategies. In essence, meticulous CIPF surveillance in water is essential for ecological preservation, public health protection, regulatory adherence, and pollution control efficacy [7].

Despite advancements in analytical techniques, such as high-performance liquid chromatography [8], turbidimetry [9], spectrophotometry [10], spectrofluorometry [11], capillary electrophoresis [12], and chemiluminescence [13], their complexity and cost remain significant impediments. Skilled operators are requisite, and the time taken to generate results can amplify expenses if errors occur during testing. To overcome these challenges, there is a pressing need for more efficient and cost-effective methods for monitoring CIPF. An electrochemical method offers simplicity, affordability, rapidity, easy handling, and miniaturization, which could favor the selective and sensitive detection of CIPF [1,14,15]. Moreover, the performance of the sensors is clearly dependent on the selection of electrode-modifying materials, which affect the selectivity, sensitivity, reproducibility, reusability, and stability of the constructed electrode [16,17]. Therefore, a huge effort has been devoted to the fabrication of appropriate platforms (individual or combination), which include metal carbides, metal nanoparticles, metal oxides, carbon nanomaterials, polymers, redox mediators, and metal–organic frameworks [18,19,20,21].

Metal carbides (MXenes) are excellent two-dimensional (2D) nanomaterials with a chemical formula M_n+1_X_n_T_x_, where M denotes transition metals (titanium, vanadium, molybdenum, and niobium), X represents either nitrogen or carbon, n stands for the values of 1–3, and T_x_ denotes the various surface terminal functional groups like hydroxyl (–OH), fluoride (–F), and oxide (=O) [22,23,24]. Among the 20 categories of confirmed MXenes, titanium carbide (Ti_3_C_2_T_x_) is a better material for application in the field of electrochemical sensors, electrocatalysis, energy conversion, and storage because of its tunable physicochemical properties, which include facile synthesis and functionalization, outstanding electrical conductivity, good mechanical strength, excellent biocompatibility, high stability, and hydrophilicity [25,26,27]. MXenes are prepared by selectively removing aluminum layers from the metallic conductive layer of Ti_3_AlC_2_ MAX phases through etching. However, the application of Ti_3_C_2_T_x_ MXene sheets directly to an electrochemical sensing, biosensing, and immunosensing platform is challenging due to their self-stacking property, resulting in poor conductivity, stability, sensitivity, and selectivity [28]. In order to address this issue, suitable conducting nanomaterials, such as metal nanoparticles (MNPs), metal oxides, polymers, etc., have been introduced into Ti_3_C_2_T_x_ MXene sheets, which increases the interlayer distance between Ti_3_C_2_T_x_ MXene sheets [29,30]. Among various MNPs, gold nanoparticles (AuNPs) are particularly advantageous in this scenario due to their excellent stability, good redox activity, high electrical conductivity, mechanical strength, biocompatibility, high surface-to-volume ratio, and chemical inertness [31,32,33]. Based on these superior properties, many research groups have incorporated AuNPs into Ti_3_C_2_T_x_ MXene (AuNPs@Ti_3_C_2_T_x_) in which AuNP solutions are simply mixed with Ti_3_C_2_T_x_ MXene [34,35,36].

In this current work, for the first time, AuNPs are embedded in Ti_3_C_2_T_x_ MXene through the chemical reaction between AuNPs and thiol-functionalized Ti_3_C_2_T_x_ MXene (SH-Ti_3_C_2_T_x_ MXene). The strong interaction between AuNPs and SH-Ti_3_C_2_T_x_ MXene can hinder the aggregation of AuNPs and endow larger electroactive surface areas. Initially, a surface hydroxyl-functionalized Ti_3_C_2_T_x_ MXene sheet is synthesized, to which 3-mercaptopropyl trimethoxysilane (MPTMS) is covalently bound through silanization to form thiolated Ti_3_C_2_T_x_ MXene sheets (SH-Ti_3_C_2_T_x_ MXene). Subsequently, AuNPs are embedded in the SH-Ti_3_C_2_T_x_ MXene to form AuNPs-S-Ti_3_C_2_T_x_ MXene. During the reaction, [AuCl_4_]^−^ anions self-assemble on the surface of the positive charge of thiol, resulting in the formation of the covalent bond of Au-S. Finally, the prepared AuNPs-S-Ti_3_C_2_T_x_ MXene nanocomposites are immobilized on the surface of a disposable integrated screen-printed carbon electrode (SPCE) to form AuNPs-S-Ti_3_C_2_T_x_ MXene/SPCEs. The resulting AuNPs-S-Ti_3_C_2_T_x_ MXene-modified electrode is employed as a working electrode for the electrochemical determination of CIPF. The prepared AuNPs-S-Ti_3_C_2_T_x_ MXene nanocomposites are characterized using field-emission scanning electron microscopy (FESEM), energy-dispersive X-ray spectroscopy (EDS), X-ray diffraction (XRD), X-ray photoelectron spectroscopy (XPS), Fourier transform infrared (FTIR) spectroscopy, and UV–visible spectroscopy. Their electrochemical properties and activity are examined and compared to those of AuNPs-Ti_3_C_2_T_x_ MXene prepared using a conventional method using cyclic voltammetry (CV), electrochemical impedance spectroscopy (EIS), differential pulse voltammetry (DPV), and linear sweep voltammetry (LSV) techniques. The sensitivity, limit of detection (LOD), and selectivity of the AuNPs-S-Ti_3_C_2_T_x_ MXene/SPCE for CIPF sensing are evaluated, and the electrochemical detection of CIPF in real milk and water samples is also investigated. Electrochemical analysis results show that the AuNPs-S-Ti_3_C_2_Tx MXene/SPCE has a higher electrochemical performance than the conventional AuNPs-Ti_3_C_2_Tx MXene/SPCE. This novel electrochemical sensor shows high sensitivity and selectivity with a wide linear range toward CIPF detection.

## 2. Materials and Methods

The disposable integrated SPCE was purchased from Metrohm DropSens (Llanera, Spain). Milk samples were gathered from a local market in Seoul, South Korea (with and without the removal of protein, fat, and other ingredients). Water samples were collected from a Dongguk University lab and the Han River in Seoul, Republic of Korea. Other chemicals and reagents, instrument details, and real sample preparations are described in the Supplementary Materials Sections S2.1–S2.3.

### 2.1. Synthesis of Thiol-Functionalized Ti_3_C_2_T_x_ MXene (SH-Ti_3_C_2_T_x_ MXene)

The synthesis procedure of Ti_3_C_2_T_X_ MXene from Ti_3_AlC_2_ MAX phases and thiol functionalization of Ti_3_C_2_T_X_ MXene are described in detail in Sections S2.4 and S2.5 of the Supplementary Materials, respectively.

### 2.2. Synthesis of Gold Nanoparticle-Embedded Thiol-Functionalized Ti_3_C_2_T_x_ MXene (AuNPs-S-Ti_3_C_2_T_x_ MXene)

AuNPs-S-Ti_3_C_2_T_x_ MXene was synthesized as follows: 0.01 g of SH-Ti_3_C_2_T_x_ MXene was dispersed in 20 mL of double distilled (DD) water (0.5 mg/mL) via ultrasonication for 2 h. Then, the freshly prepared aqueous solution of HAuCl_4_ (5 mM; 0.5 mL) was injected drop by drop to a magnetic stirred (400 rpm) suspension of SH-Ti_3_C_2_T_x_ MXene, which was constantly stirred (400 rpm) for another 30 min to facilitate the chelation of [AuCl_4_]^−^ ions with the thiol (–SH) groups on SH-Ti_3_C_2_T_x_ MXene. Subsequently, a fresh aqueous solution of NaBH_4_ (0.1 M; 0.2 mL) was immediately injected into the above colloidal solution, and the color of the colloidal solution suddenly changed from pale yellow to purplish red, which indicates the reduction of Au^3+^ to Au^0^. The stability of AuNPs-S-Ti_3_C_2_T_x_ MXene in solution was outstanding, and no aggregation was observed even up to 90 days. For comparison, AuNPs-Ti_3_C_2_T_x_ MXene was also prepared using the same experimental procedure as mentioned above from Ti_3_C_2_T_x_ MXene instead of SH-Ti_3_C_2_T_x_ MXene.

### 2.3. Fabrication of AuNPs-S-Ti_3_C_2_T_x_ MXene-Modified SPCE

**Figure 1:**
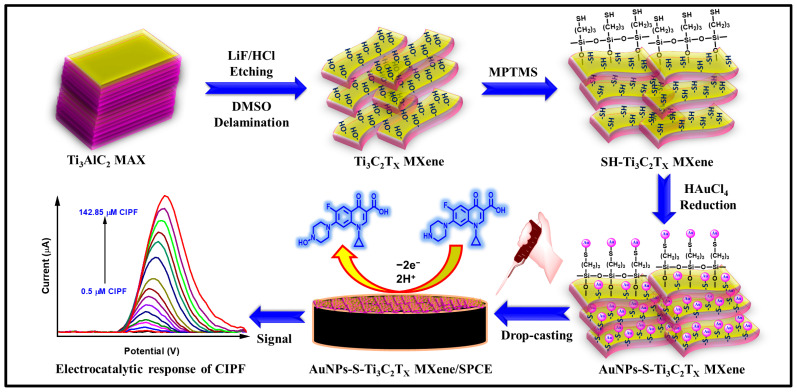
Preparation of AuNPs-S-Ti_3_C_2_T_x_ MXene/SPCE and schematic electrochemical detection of CIPF using the nanocomposite-modified SPCE.

Before electrode modifications, the bare SPCE was thoroughly washed with DD water and dried at ambient temperature. Different amounts of AuNPs-S-Ti_3_C_2_T_x_ MXene (2 µL increment; ~0.5 mg/mL) have been immobilized by drop-casting on the surface of the pre-cleaned integrated SPCE and then allowed to dry at 25 °C. The AuNPs-S-Ti_3_C_2_T_x_ MXene-modified electrode was thoroughly washed with a 0.1 M phosphate buffer solution (PBS; pH 7.0) (to remove loosely bound molecules or particles) and dried at room temperature before electrochemical investigations. Finally, the built-up electrode was tested for CIPF detection using CV (Figure S1). The fabrication of AuNPs-S-Ti_3_C_2_T_x_ MXene-modified SPCE and the schematic electrochemical CIPF sensor are presented in Figure 1. For comparison, Ti_3_C_2_T_x_ MXene/SPCE, SH-Ti_3_C_2_T_x_ MXene/SPCE, and AuNPs-Ti_3_C_2_T_x_ MXene/SPCE were also fabricated under the same experimental conditions.

## 3. Results and Discussion

### 3.1. Surface Morphological Investigation of AuNPs-S-Ti_3_C_2_T_x_ MXene

**Figure 2:**
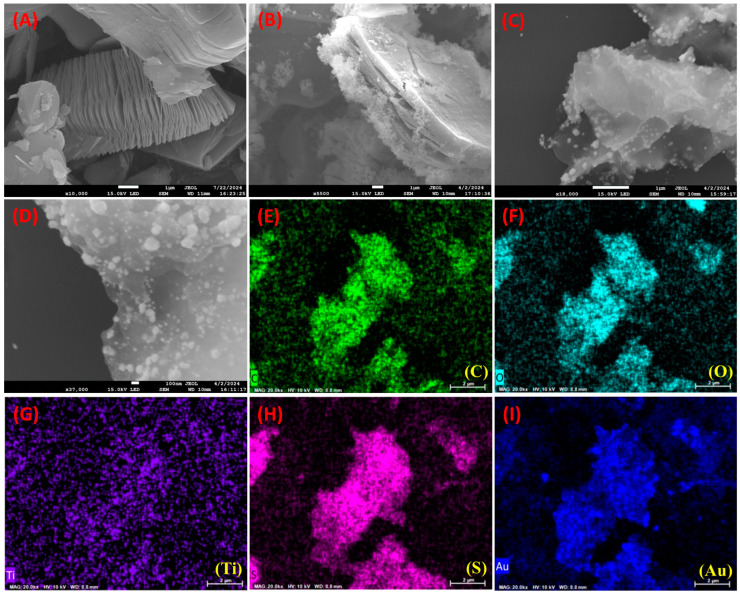
FESEM images of (**A**) Ti_3_C_2_T_x_ MXene, (**B**) SH-Ti_3_C_2_T_x_ MXene, and (**C**,**D**) AuNPs-S-Ti_3_C_2_T_x_ MXene under different magnifications. Elemental mapping images of AuNPs-S-Ti_3_C_2_T_x_ MXene: (**E**) carbon, (**F**) oxygen, (**G**) titanium, (**H**) sulfur, and (**I**) gold.

The surface morphology structures of the synthesized Ti_3_C_2_T_x_ MXene, SH-Ti_3_C_2_T_x_ MXene, and AuNPs-S-Ti_3_C_2_T_x_ MXene nanocomposites were characterized by FESEM, and the results are displayed in Figure 2. Unetched pristine Ti_3_AlC_2_ particles were shown as blocky MAX phases [22]. After etching off the aluminum layer between blocky Ti_3_AlC_2_ with LiF and HCl, the nanosheet structure of Ti_3_C_2_T_x_ was observed, as presented in Figure 2A. The EDS analysis and the corresponding elemental distribution of Ti_3_C_2_T_x_ MXene sheets are shown in Figure S2, which can be witnessed due to the elimination of the aluminum layer and the transformation of Ti_3_AlC_2_ MAX phases to Ti_3_C_2_T_x_ MXene sheets [22]. Further, the thiol-functionalized Ti_3_C_2_T_x_ MXene displayed variations in surface roughness that can be observed on the surface of Ti_3_C_2_T_x_ MXene (Figure 2B). EDS and elemental mapping results are presented in Figure S3, which confirms the thiol functionalization. Additionally, as seen in Figure 2C,D (under different magnifications), many nanoparticles can be vividly noticed on the surface of AuNPs-S-Ti_3_C_2_T_x_ MXene, confirming the successful decoration of Ti_3_C_2_T_x_ MXene sheets with AuNPs [37]. Moreover, a relatively dense population of AuNPs with minimum particle aggregation was observed, which could be ascribed to the strong interaction between AuNPs and thiol groups. Figure S4 depicts the EDS analysis, and the corresponding elemental mapping images with uniform distribution of C, O, Ti, S, and Au elements are shown in Figure 2E–I.

### 3.2. Structural Analysis of AuNPs-S-Ti_3_C_2_T_x_ MXene

**Figure 3:**
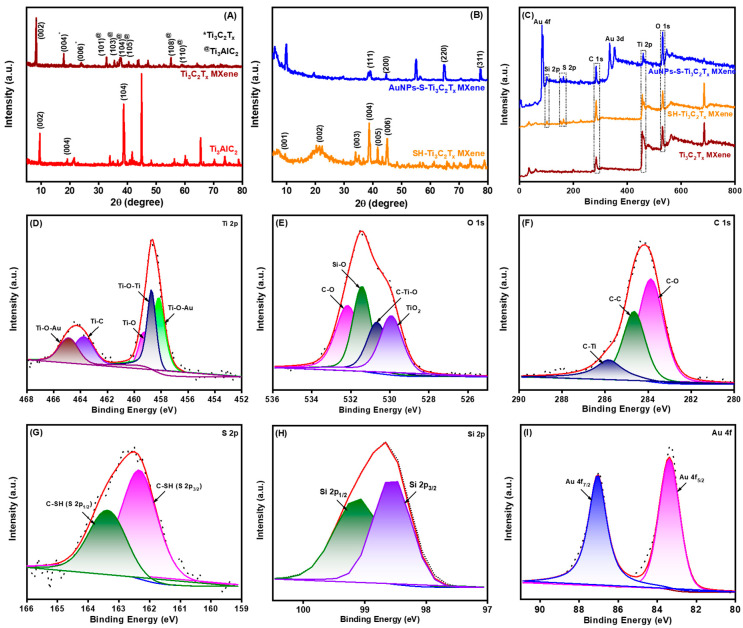
(**A**,**B**) XRD and (**C**) high-resolution XPS survey spectra of Ti_3_C_2_T_x_ MXene, SH-Ti_3_C_2_T_x_ MXene, and AuNPs-S-Ti_3_C_2_T_x_ MXene. Core level scan of the (**D**) Ti 2p, (**E**) C 1s, (**F**) O 1s, (**G**) S 2p, (**H**) Si 2p, and (**I**) Au 4f for AuNPs-S-Ti_3_C_2_T_x_ MXene.

The XRD method was used to analyze the purities and crystal structures of the commercial Ti_3_AlC_2_ MAX precursor and the prepared samples (Ti_3_C_2_T_x_ MXene, SH-Ti_3_C_2_T_x_ MXene, and AuNPs-S-Ti_3_C_2_T_x_ MXene). As shown in Figure 3A,B, the XRD pattern of the Ti_3_AlC_2_ MAX precursor had numerous peaks, and the major peaks at 9.6°, 19.27°, and 38.85° correspond to the (002), (004), and (104) crystallographic planes, respectively. After etching, the characteristic XRD pattern of strong 9.6° (002) and 19.27° (004) peaks of the Ti_3_AlC_2_ MAX phases was shifted to a lower angle of 8.32° and 17.93° due to the expansion of the interlayer distance between the crystal planes [38]. In addition, the strongest peak at 38.85° (104) of Ti_3_AlC_2_ MAX completely vanished in both Ti_3_C_2_T_x_ MXene and SH-Ti_3_C_2_T_x_ MXene, indicating the transformation of Ti_3_AlC_2_ MAX phases to the Ti_3_C_2_T_x_ MXene by aluminum layer elimination [29]. In addition, the characteristic planes of (006), (101), (103), (105), (108), and (110) Ti_3_C_2_T_x_ MXene notably decreased, indicating that the substantial reduction in the crystalline nature of the bulk-stacked MAX powder. After the modification of Ti_3_C_2_T_x_ MXene with −SH and AuNPs, the standard planes of Ti_3_C_2_T_x_ MXene were significantly decreased, which can be attributed to its small diffraction peak intensity and low contents. Moreover, the XRD spectra of SH-Ti_3_C_2_T_x_ MXene exhibited six noticeable planes that are listed as (001), (002), (003), (004), (005), and (006), corresponding to the layered structure of sheets. On the other hand, in the XRD of AuNPs-S-Ti_3_C_2_T_x_ MXene, the peaks at 38.6°, 44.3°, 64.5°, and 77.4° are attributed to the (111), (200), (220), and (311) lattice planes of AuNPs, respectively, confirming the in situ formation of AuNPs [31]. The average size of the AuNPs was calculated to be 20–120 nm using Scherer’s equation (L = 0.9λ/β (2θ) × cosθ_max_). These results indicate that AuNPs-S-Ti_3_C_2_T_x_ MXene nanocomposite was effectively synthesized.

The surface chemical components and electronic state of the elements in Ti_3_C_2_T_x_ MXene, SH-Ti_3_C_2_T_x_ MXene, and AuNPs-S-Ti_3_C_2_T_x_ MXene were characterized using XPS. As shown in Figure 3C, the XPS survey spectra of the above three samples portrayed that C 1s (284.4 eV), O 1s (531.8 eV), and Ti 2p (458.8 eV) signals appeared in all the samples. The clear S 2p (163.2 eV) signal was observed in SH-Ti_3_C_2_T_x_ MXene and AuNPs-S-Ti_3_C_2_T_x_ MXene nanocomposites, whereas the Au 4f (82.2 eV) signal appeared only in AuNPs-S-Ti_3_C_2_T_x_ MXene nanocomposite. These XPS survey spectra confirmed the elimination of the aluminum layer in all samples. The individual elemental peaks, along with their deconvoluted peaks, originated from the presence of underlying chemical species for Ti_3_C_2_T_x_ MXene (Figure S5), SH-Ti_3_C_2_T_x_ MXene (Figure S6), and AuNPs-S-Ti_3_C_2_T_x_ MXene (Figure 3D–I). The Ti 2p spectrum of the AuNPs-S-Ti_3_C_2_T_x_ MXene nanocomposite was divided into Ti 2p_1/2_ and Ti 2p_3/2_ spin-orbit doublets. The Ti 2p was deconvoluted into four valence state peaks after fitting (Figure 3D): the fitting peak at 459.2 eV ascribed to the Ti–O bonds, the fitting peaks at 464.7 and 457.2 eV corresponding to the Ti–O–Au bonds, the fitting peak at 458.8 eV ascribed to the Ti–O–Ti bonds, and the fitting peak at 463.7 eV corresponding to the Ti–C bonds [39]. The titanium in the AuNPs-S-Ti_3_C_2_T_x_ MXene nanocomposite contained several valence states, and the chemical oxidation process caused Ti(II) and Ti(III) to transform into Ti(IV). In addition, the bulky peak area of TiO_2_ indicated the partial oxidation of titanium elements during ultrasonication exfoliation. This result was further evidenced by the occurrence of a Ti–O bond at 531.8 eV in the O 1s XPS spectrum. The O 1s XPS spectrum of AuNPs-S-Ti_3_C_2_T_x_ MXene nanocomposite was fit by four peaks (Figure 3E): the fitting peaks at 532.2 eV, 531.5 eV, 530.6 eV, and 529.8 eV corresponding to the C–O, Si–O, C–Ti–O, and TiO_2_ bonds, respectively [40]. Further, the C 1s spectrum of the AuNPs-S-Ti_3_C_2_T_x_ MXene nanocomposite was deconvoluted into three valence state peaks (Figure 3F): the narrow fitting peaks at 285.5 eV, 284.6 eV, and 283.8 eV ascribed to the C–Ti, C–C, and C–O bonds, respectively [40,41]. The huge C–C components (~41% of C 1s spectrum) indicated the sheet-like structure of the prepared Ti_3_C_2_T_x_ MXene, offering strong interaction with thiol. Furthermore, the S 2p XPS spectrum of AuNPs-S-Ti_3_C_2_T_x_ MXene nanocomposite (Figure 3G) was deconvoluted into S 2p_1/2_ and S 2p_3/2_ (C–SH) spin-orbit doublets, along with the satellite peaks at a binding energy of 163.4 eV and 162.3 eV, respectively. The Si 2p XPS spectrum of AuNPs-S-Ti_3_C_2_T_x_ MXene was deconvoluted into two valence state peaks (Figure 3I): the fitting peaks at 99.1 eV and 98.9 eV characteristic of the Si 2p_1/2_ and Si 2p_3/2_ bonds, respectively. Finally, the Au 4f spectrum of the AuNPs-S-Ti_3_C_2_T_x_ MXene nanocomposite displayed two major deconvoluted valence state peaks related to the zero-valent gold (Au) element with a strong binding energy of 87.1 eV and 83.3 eV, and the binding energy separation was 3.8 eV (Figure 3H). The binding energy corresponds to the Au 4f_7/2_ and Au 4f_5/2_ bonds [42]. The XPS results indicated the massive adhesion and compatibility between AuNPs and SH-Ti_3_C_2_T_x_ MXene, illustrating the formation of a well-integrated nanocomposite structure. The surface-bound interfacial contact between AuNPs and Ti_3_C_2_T_x_ MXene could play a significant role in the overall nanocomposite stability, conductivity, and performance, ensuring effective electron transfer during its utilization as a sensing platform.

### 3.3. FTIR and UV–Visible Analysis of AuNPs-S-Ti_3_C_2_T_x_ MXene

**Figure 4:**
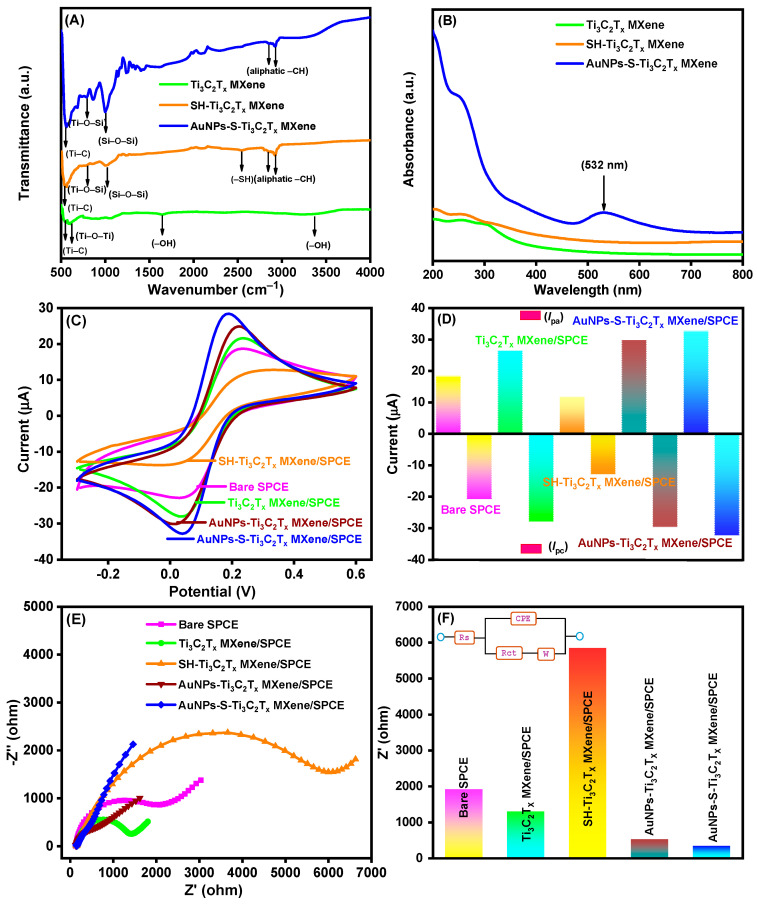
(**A**) FTIR and (**B**) UV–visible absorption spectra of Ti_3_C_2_T_x_ MXene (green curve), SH-Ti_3_C_2_T_x_ MXene (orange curve), and AuNPs-S-Ti_3_C_2_T_x_ MXene (blue curve). (**C**) CV curves (**D**) peak currents (*I*_pa_ and *I*_pc_) for various modified SPCEs. (**E**) EIS curves and (**F**) resistance (*R*_ct_) for various modified SPCEs. Electrodes: bare SPCE (pink curve), Ti_3_C_2_T_x_ MXene/SPCE (green curve), SH-Ti_3_C_2_T_x_ MXene/SPCE (orange curve), AuNPs-Ti_3_C_2_T_x_ MXene/SPCE (maroon curve), and AuNPs-S-Ti_3_C_2_T_x_ MXene/SPCE (blue curve); electrolyte: 0.1 M KCl containing 2.5 mM [Fe(CN)_6_]^3−^ and 2.5 mM [Fe(CN)_6_]^4−^.

FTIR spectroscopy was used to examine the functional groups in Ti_3_C_2_T_x_ MXene, SH-Ti_3_C_2_T_x_ MXene, and AuNPs-S-Ti_3_C_2_T_x_ MXene nanocomposites, as portrayed in Figure 4A. The spectrum of Ti_3_C_2_T_x_ MXene shows absorption peaks at 1637 cm^−1^ and 3337 cm^−1^ stretching vibration of hydroxyl groups (–OH) introduced in the Ti_3_C_2_T_x_ MXene sheets [43]. The strong absorption bands at 546 cm^−1^ and 847 cm^−1^ are characteristic of the Ti–C and Ti–O–Ti bonds in Ti_3_C_2_T_x_ MXene sheets, respectively [43]. Further, covalent immobilization of MPTMS on Ti_3_C_2_T_x_ MXene sheets through silanization diminished the absorption bands of the hydroxyl group, which is because the –OH groups underwent condensation with the methoxy (–OCH_3_) group of MPTMS. In addition, the new peaks observed at 1015 cm^−1^ and 2548 cm^−1^ are characteristic of the vibration of Si–O–Si and –SH, respectively, and the new bands at 2918 cm^−1^ and 2983 cm^−1^ are related to stretching of the aliphatic –CH groups of MPTMS [44]. Another new peak at 793 cm^−1^, attributed to Ti–O–Si bonds [45], reconfirms the condensation between Ti_3_C_2_T_x_ MXene and MPTMS. Additional AuNP decoration diminished the free terminal –SH stretching vibration while retaining other groups. The outcomes prove that AuNPs were successfully attached to Ti_3_C_2_T_x_ MXene through MPTMS.

The decoration of AuNPs over Ti_3_C_2_T_x_ MXene was also investigated using UV–visible spectroscopy. The UV–visible absorption spectra of Ti_3_C_2_T_x_ MXene, SH-Ti_3_C_2_T_x_ MXene, and AuNPs-S-Ti_3_C_2_T_x_ MXene with a concentration of 0.5 mg/mL in DD water are portrayed in Figure 4B. The MXenes exhibited absorption in the UV region, which may correspond to the band-gap energy of the oxidized MXenes [46]. AuNPs-S-Ti_3_C_2_T_x_ MXene showed a peak at 532 nm due to the surface plasmon resonance characteristic of AuNPs, which proves the presence of AuNPs in the Ti_3_C_2_T_x_ MXene sheet [31].

### 3.4. Electrochemical Behavior of the AuNPs-S-Ti_3_C_2_T_x_ MXene-Modified SPCE

The electrochemical property of the modified SPCEs was assessed simply by CV. Figure 4C shows the cyclic voltammograms of the bare SPCE, Ti_3_C_2_T_x_ MXene/SPCE, SH-Ti_3_C_2_T_x_ MXene/SPCE, AuNPs-Ti_3_C_2_T_x_ MXene/SPCE, and AuNPs-S-Ti_3_C_2_T_x_ MXene/SPCE recorded in 0.1 M KCl in the presence of 2.5 mM [Fe(CN)_6_]^3−^ and 2.5 mM [Fe(CN)_6_]^4−^ at a scan rate of 50 mV/s. For comparison, an AuNPs-Ti_3_C_2_T_x_ MXene/SPCE was fabricated without thiol functionalization under the same conditions as the AuNPs-S-Ti_3_C_2_T_x_ MXene/SPCE. The CV signal of the bare SPCE (pink curve) in ferro/ferric redox solution showed well-defined anodic and cathodic peak currents (*I*_pa_ and *I*_pc_). Modification of the bare SPCE with Ti_3_C_2_T_x_ MXene (Ti_3_C_2_T_x_ MXene/SPCE) increased the peak currents notably (green curve), which could be due to the massive surface area of Ti_3_C_2_T_x_ MXene. However, the thiol modification of Ti_3_C_2_T_x_ MXene/SPCE decreased the peak currents prominently (orange curve). This may be because of the formation of an insulating layer of non-conductive MPTMS on the surface of the modified electrode. After decoration with AuNPs, the resultant AuNPs-Ti_3_C_2_T_x_ MXene/SPCE (maroon curve) and the AuNPs-S-Ti_3_C_2_T_x_ MXene/SPCE (blue curve) showed higher redox peak currents than the Ti_3_C_2_T_x_ MXene/SPCE. This is attributed to the improvement in surface areas and electrical conductivity by AuNPs [47]. Noticeably, the AuNPs-S-Ti_3_C_2_T_x_ MXene/SPCE was found to display the highest current response among all the types of modified electrodes, proving the effectiveness of the thiol functionalization. This may be because the strong interaction between AuNPs and SH-Ti_3_C_2_T_x_ MXene hinders the aggregation of AuNPs and endows larger electroactive surface areas. The electroactive surface areas of the bare SPCE and modified SPCEs are presented in detail in Section S3.1 of the Supplementary Materials. In Figure 4D, the bar diagram of the *I*_pa_ and *I*_pc_ responses of the five different SPCEs is shown.

The electrochemical properties of the bare and modified SPCEs were also examined using EIS, encompassing the kinetics of electron-transfer reactions and the electrochemical interfacial properties. Figure 4E portrays the EIS curves for different electrodes (bare SPCE, Ti_3_C_2_T_x_ MXene/SPCE, SH-Ti_3_C_2_T_x_ MXene/SPCE, AuNPs-Ti_3_C_2_T_x_ MXene/SPCE, and AuNPs-S-Ti_3_C_2_T_x_ MXene/SPCE) using Nyquist plots. In order to fit the Randles equivalent circuit, the following elements were selected in the circuit: a charge transfer resistance (*R*_ct_), a Warburg impedance (*W*), an electrolyte solution resistance (*R*_s_), and a constant phase element (*CPE*) representing the non-ideal or double-layer capacitance. The typical EIS plots of the sensor platform were obtained in 0.1 M KCl containing 2.5 mM [Fe(CN)_6_]^3−^ and 2.5 mM [Fe(CN)_6_]^4−^ over a frequency range commencing from 100 kHz to 0.01 Hz. According to the Randles equivalent circuit, the EIS spectrum of the bare SPCE displayed a dispersed semicircle (*R*_ct_ = 1912 Ω), revealing the slow interfacial electron transfer ability occurring at the bare SPCE. After immobilization of Ti_3_C_2_T_x_ MXene on the SPCE, the *R*_ct_ value decreased to 1289 Ω due to the tendency of Ti_3_C_2_T_x_ MXene sheet to enhance electron transfer ability. On a subsequent modification with MPTMS on Ti_3_C_2_T_x_ MXene, the *R*_ct_ value increased to 5850 Ω. This is due to the formation of an insulating layer of non-conductive MPTMS on the modified electrode surface. Further, upon decoration with AuNPs, the *R*_ct_ value considerably decreased to 383 Ω (AuNPs-Ti_3_C_2_T_x_ MXene/SPCE) and 331 Ω (AuNPs-S-Ti_3_C_2_T_x_ MXene/SPCE), indicating that the combination of a Ti_3_C_2_T_x_ MXene sheet and AuNPs enhances electrochemical properties at the modified electrode [47]. The EIS results correlated well with the CV results during the consecutive electrode modifications. The *R*_ct_ values for the bare and modified SPCE are displayed in Figure 4F, and the corresponding Randles equivalent circuit is shown in the inset of Figure 4F.

### 3.5. Electrocatalytic Oxidation of CIPF with the AuNPs-S-Ti_3_C_2_T_x_ MXene-Modified SPCE

**Figure 5:**
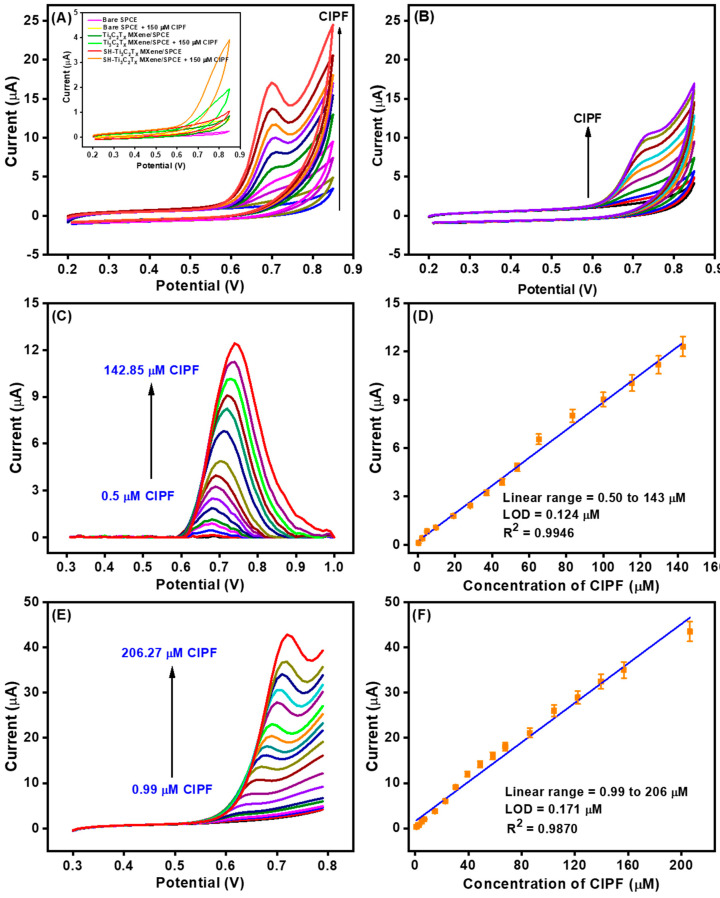
Cyclic voltammograms of (**A**) AuNPs-S-Ti_3_C_2_T_x_ MXene/SPCE and (**B**) AuNPs-Ti_3_C_2_T_x_ MXene/SPCE with increasing concentrations of CIPF (each 25 µM of CIPF) in 0.1 M PBS (pH 7.0) at a scan rate of 50 mV/s. Inset to (**A**): cyclic voltammograms of the bare SPCE, Ti_3_C_2_T_x_ MXene/SPCE, and SH-Ti_3_C_2_T_x_ MXene/SPCE in the absence and presence of 150 µM CIPF. (**C**) DPV (baseline correction) and (**E**) LSV current response of the AuNPs-S-Ti_3_C_2_T_x_ MXene/SPCE with different concentrations of CIPF in 0.1 M PBS (pH 7.0). (**D**) DPV and (**F**) LSV: corresponding calibration plot for the determination of CIPF.

The electrocatalytic responses of the AuNPs-S-Ti_3_C_2_T_x_ MXene/SPCE towards nonenzymatic detection of CIPF were investigated in 0.1 M PBS using CV with a potential range from 0.2 to 0.85 V. First, the effects of a scan rate and pH were examined as described in Sections S3.2 and S3.3 of the Supplementary Materials, respectively. It was revealed that the CIPF oxidation at the AuNPs-S-Ti_3_C_2_T_x_ MXene/SPCE was an adsorption-controlled process (Figure S7), and the optimal pH was 7.0 (Figure S8). In contrast to the AuNPs-S-Ti_3_C_2_T_x_ MXene/SPCE, the bare SPCE, Ti_3_C_2_T_x_ MXene/SPCE, and SH-Ti_3_C_2_T_x_ MXene/SPCE displayed an infirm catalytic response towards the oxidation in the presence and absence of 150 μM CIPF (inset of Figure 5A). The electrocatalytic response of the AuNPs-S-Ti_3_C_2_T_x_ MXene/SPCE increased linearly with the repeated addition of CIPF (each addition 25 μM), indicating the uniform electrocatalytic oxidation of CIPF at the surface of the modified electrode. During the anodic scan, the –NH group of CIPF is electrochemically oxidized to form a –N–OH derivative. These observations confirm that the improved *I*_pa_ response to CIPF at the AuNPs-S-Ti_3_C_2_T_x_ MXene/SPCE is due to the enhanced electroactive area and conductivity of AuNPs. For comparison, the AuNPs-Ti_3_C_2_T_x_ MXene/SPCE was also examined under the same conditions (Figure 5B). Compared to the AuNPs-S-Ti_3_C_2_T_x_ MXene/SPCE, the CIPF oxidation peak was observed at a more positive potential, and the anodic peak current was much lower at the same CIPF concentrations. This clearly manifests the advantages of the thiol-functionalized Ti_3_C_2_T_x_ MXene introduced in this work.

The sensitivity and limit of detection (LOD) of the AuNPs-S-Ti_3_C_2_T_x_ MXene/SPCE for CIPF sensing were determined using DPV and LSV techniques. Figure 5C,E depict the DPV and LSV responses of the AuNPs-S-Ti_3_C_2_T_x_ MXene/SPCE towards spiking different concentrations of CIPF under the optimized operating circumstances, respectively. As expected, the AuNPs-S-Ti_3_C_2_T_x_ MXene/SPCE displayed a linear, uniform, and stable electrocatalytic response towards CIPF, which signifies the rapid electron transfer kinetics, excellent sensitivity, and consistent electrocatalytic activity of the newly devised electrode. The linear regression equation between the CIPF concentration and oxidation peak current was obtained as *I* (μA) = 0.0863 (μM) + 0.2203 (R^2^ = 0.9946) (DPV, Figure 5D) and *I* (μA) = 0.2182 (μM) + 1.5584 (R^2^ = 0.9870) (LSV, Figure 5F). Moreover, the newly devised electrode exhibited exceptional electrocatalytic response towards CIPF over a wide concentration range from 0.50 to 143 μM (DPV) and from 0.99 to 206 μM (LSV). The sensitivity was calculated from the slope (*m*) of the calibration plot, and LOD was calculated using the formula 3*σ*/*m* (S/N = 3), where *σ* represents the standard deviation of the blank experiment. The sensitivity and LOD using DPV were found to be 0.0863 μA/μM and 0.124 μM, respectively, and those using LSV were 0.2182 μA/μM and 0.171 μM, respectively. The performance of the new CIPF sensor was better than or comparable with that of recently published nonenzymatic electrochemical sensors in terms of linear range, LOD, and sensitivity (Table 1). Thus, the outstanding electrocatalytic oxidation performance arises from the enhanced electroactive area and electrical conductivity by AuNPs strongly bound to SH-Ti_3_C_2_T_x_ MXene. The excellent electrocatalytic sensing behavior is favorable for the detection of CIPF in real samples.

### 3.6. Selectivity, Stability, and Reproducibility of the AuNPs-S-Ti_3_C_2_T_x_ MXene/SPCE

**Figure 6:**
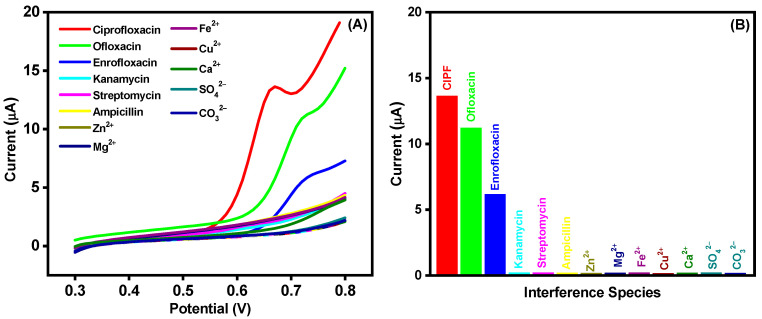
(**A**) LSV peak current response to 50 µM CIPF and electroactive interferents (25 µM ofloxacin and enrofloxacin, 500 µM ampicillin, kanamycin, streptomycin, Zn^2+^, Mg^2+^, Fe^2+^, Cu^2+^, Ca^2+^, SO_4_^2−^, and CO_3_^2−^). (**B**) Current response columnar diagram of the examined interferents compared with CIPF. Electrolyte: 0.1 M PBS (pH 7.0) at a scan rate of 50 mV/s.

Selectivity of nonenzymatic sensors is always essential for real-time monitoring, and the selectivity experiments of the AuNPs-S-Ti_3_C_2_T_x_ MXene/SPCE were conducted with the LSV technique (Figure 6A). The most common electroactive analytes co-existing in biological and environmental samples (ofloxacin, enrofloxacin, ampicillin, kanamycin, streptomycin, Zn^2+^, Mg^2+^, Fe^2+^, Cu^2+^, Ca^2+^, SO_4_^2−^, and CO_3_^2−^) were used as interferents and spiked at regular intervals into 0.1 M PBS (pH 7.0) in addition to CIPF. It could be observed that, after injection of CIPF (50 μM), the oxidation current increased evidently. Ofloxacin and enrofloxacin (25 μM) showed 28% and 16% interference, respectively, whereas ampicillin, kanamycin, streptomycin, Zn^2+^, Mg^2+^, Fe^2+^, Cu^2+^, Ca^2+^, SO_4_^2−^, and CO_3_^2−^ with a tenfold higher concentration (500 μM) displayed no current or negligible response (Figure 6B). The excellent selectivity of the AuNPs-S-Ti_3_C_2_T_x_ MXene/SPCE reveals that it is a promising sensor for the determination of CIPF.

The stability of the prepared AuNPs-S-Ti_3_C_2_T_x_ MXene-modified electrode was also evaluated by constant potential cycling at a scan rate of 50 mV/s. Figure S9 exhibits 50 continuous cyclic voltammograms of AuNPs-S-Ti_3_C_2_T_x_ MXene/SPCE in 0.1 M PBS (pH 7.0) containing 100 μM CIPF. There were no apparent differences in the peak potential, and the oxidation current showed a slight decrease during these repeated cycles, revealing that the proposed sensor retains 72% of its initial current response at the end of the 50th cycle. Additionally, the reproducibility of the developed CIPF sensor was investigated with five fresh AuNPs-S-Ti_3_C_2_T_x_ MXene/SPCEs fabricated using the same procedure. Their electrocatalytic oxidation current responses on spiking of 150 μM CIPF (CV technique, 0.1 M PBS) are shown in Figure S10A, and the relative standard deviation (RSD) attained among the constructed sensors was 11.7%, representing reasonable electrode-to-electrode reproducibility (Figure S10B).

### 3.7. Analysis of Real Samples

To explore the practical applicability of the developed nonenzymatic sensor, the detection of CIPF in real milk and water samples was attempted. Milk samples were obtained from a local market in Seoul, South Korea, and labeled samples #1 and #2 (with and without the removal of protein, fat, and other ingredients, respectively). Water samples were collected from a Dongguk University lab and the Han River in Seoul, South Korea, and labeled samples #3 and #4, respectively. This study was performed under optimal conditions, wherein the milk and water samples were diluted 25-fold using 0.1 M PBS (pH 7.0). Known concentrations of CIPF were injected into the milk and water samples, and the CIPF concentrations in the samples were measured with the AuNPs-S-Ti_3_C_2_T_x_ MXene/SPCE sensor using LSV (Figure S11). It was found that the sensor recoveries in the milk and water samples ranged from 91.9 to 109.6% (milk samples) and from 89.7 to 110.3% (water samples) with low RSDs of <4% (Table S1). These outcomes demonstrate that the sensor developed in this work can be reliably used for the quantification of CIPF in real samples.

## 4. Conclusions

For the first time, the thiol functionalization of MXene has been performed to embed gold nanoparticles in Ti_3_C_2_T_x_ MXene. AuNPs were deposited in situ on SH-Ti_3_C_2_T_x_ MXene, and the resultant AuNPs-S-Ti_3_C_2_T_x_ MXene nanocomposite was utilized as an electrochemical platform for the sensitive determination of CIPF. Owing to the strong interaction between AuNPs and SH-Ti_3_C_2_T_x_ MXene, the AuNPs-S-Ti_3_C_2_T_x_ MXene/SPCE showed higher electrochemical performances towards CIPF oxidation than the conventional AuNPs-Ti_3_C_2_T_x_ MXene/SPCE. This modified electrode exhibited a wide linear concentration range from 0.50 to 143 μM (DPV) and from 0.99 to 206 μM (LSV) with a low LOD of 0.124 μM (DPV) and 0.171 μM (LSV). The developed AuNPs-S-Ti_3_C_2_T_x_ MXene/SPCE sensor also showed high selectivity. Finally, the developed portable sensor was successfully employed for the quantification of CIPF in real milk and water samples, which makes it more reliable for practical applications.

## Figures and Tables

**Table 1:** Comparison between the different modified electrodes for the determination of CIPF with proposed techniques.

Electrodes	Method	Linear Range (µM)	LOD (µM)	Sensitivity (µA/µM)	Samples	Ref.
MIP/rGO/GCE	DPV	0.001–0.5	0.00005	5.78	Water	[6]
NH_2_-UiO-66/RGO	ASV	0.02–1	0.00667	10.86	Water	[14]
TiO_2_/PB/AuNPs/CMK-3/Nafion/GE	CV	1–10	0.108	15.93	Water	[15]
Cu/Ce-MOF/NZP/CPE	DPV	0.75–100	0.142	1.29	Milk, urine, and water	[19]
rGO-SnO_2_/SPE	SWV	30–100	2.03	9.348	Water and milk	[20]
MWCNT/MoS_2_/CS	DPV	0.5–1200	0.16	-	Water	[48]
Ru-Cu-TMA/GCE	DPV	2.5–100	0.00329	0.0524	Water	[49]
ChCl/CPE	SWV	0.005–200	0.00036	-	Water	[50]
PBE	DPV	9.90–220	4.96	-	Milk and honey	[51]
ERGO/PANI/PARS/SPCE	LSV	0.01–69.8	0.0021	0.4833	Milk	[52]
Ag-B-CD/GCE	DPV	0.0001–0.05	0.000028	-	Water	[53]
AuNPs-S-Ti_3_C_2_T_x_ MXene/SPCE	DPV LSV	0.5–143 0.99–206	0.124 0.171	0.0863 0.2182	Milk and water	This work

## Data Availability

The data are contained within the article or Supplementary Materials.

## Supplementary Materials

The following supporting information can be downloaded at https://www.mdpi.com/article/10.3390/nano14201655/s1: Supplementary Section S2.1: Chemicals and reagents. Section S2.2: Apparatus and instrumentation. Section S2.3: Real sample preparation. Section S2.4: Ti_3_C_2_T_x_ MXene synthesis. Section S2.5: SH-Ti_3_C_2_T_x_ MXene synthesis. Section S3.1: Electroactive surface area calculation. Section S3.2: Effect of scan rate. Section S3.3. Effect of pH. Supplementary Table S1: Real sample analysis. Supplementary Figure S1: Drop volume optimization of AuNPs-S-Ti_3_C_2_T_x_ MXene. Figure S2: EDS and elemental analysis of Ti_3_C_2_T_x_ MXene. Figure S3: EDS and elemental analysis of SH-Ti_3_C_2_T_x_ MXene. Figure S4: EDS analysis of AuNPs-S-Ti_3_C_2_T_x_ MXene. Figure S5: XPS spectra of Ti_3_C_2_T_x_ MXene. Figure S6: XPS spectra of SH-Ti_3_C_2_T_x_ MXene. Figure S7: Effect of scan rate of AuNPs-S-Ti_3_C_2_T_x_ MXene/SPCE. Figure S8: Effect of pH AuNPs-S-Ti_3_C_2_T_x_ MXene/SPCE. Figure S9: Cyclic stability of the prepared AuNPs-S-Ti_3_C_2_T_x_ MXene/SPCE modified electrode. Figure S10: Reproducibility of the developed sensor. Figure S11: Real sample analysis of milk and water samples. References [17,54,55,56,57] are cited in the Supplementary Materials.
