# Supplementary material for: Gold Nanoparticle-Embedded Thiol-Functionalized Ti3C2Tx MXene for Sensitive Electrochemical Sensing of Ciprofloxacin
Source: Nanomaterials (Basel). 2024 Oct 15;14(20):1655. doi: 10.3390/nano14201655 (PMC11510598; doi:10.3390/nano14201655)
Supplement: Supplementary file 1 [file nanomaterials-14-01655-s001.zip › nanomaterials-3257839-supplementary.pdf]

Supplementary Materials

Gold Nanoparticle-Embedded Thiol-Functionalized $Ti_3C_2T_x$ MXene for Sensitive Electrochemical Sensing of Ciprofloxacin

Mari Elancheziyan, Manisha Singh and Keehoon Won *

Department of Chemical and Biochemical Engineering, College of Engineering, Dongguk University-Seoul,
30 Pildong-ro 1-gil, Jung-gu, Seoul 04620, Republic of Korea; cheziyan@dongguk.edu (M.E.);
manisha@dgu.ac.kr (M.S.)
* Correspondence: keehoon@dongguk.edu

2. Materials and Methods

2.1. Chemicals and Reagents

Titanium aluminium carbide (Ti_3AlC_2), 3-mercaptopropyl trimethoxysilane (MPTMS), lithium fluoride (LiF), hydrochloric acid (HCl), gold(III) chloride trihydrate ($HAuCl_4 \cdot 3H_2O$), potassium ferricyanide {$K_3[Fe(CN)_6]$}, potassium ferrocyanide {$K_4[Fe(CN)_6]$}, sodium borohydride ($NaBH_4$), dimethyl sulfoxide (DMSO), ethanol, acetonitrile, toluene, sodium dihydrogen phosphate (NaH_2PO_4), disodium hydrogen phosphate (Na_2HPO_4), sodium hydroxide (NaOH), ciprofloxacin (CIPF), enrofloxacin, ofloxacin, kanamycin, ampicillin, streptomycin were obtained from Sigma-Aldrich (USA). All the chemicals and reagents were used without further purification. The analytes and electrolytes were prepared in double distilled (DD) water and stored at 4 ºC for subsequent usage.

2.2. Apparatus and Instrumentation

The surface morphology of $Ti_3C_2T_x$ MXene, SH-$Ti_3C_2T_x$ MXene, and AuNPs-S-$Ti_3C_2T_x$ MXene were examined by field emission scanning electron microscopy (FESEM, ultra plus FE Zeiss). Further, the elemental mapping and distribution of prepared nanomaterials were performed from high-angle annular dark-field scanning transmission electron microscopy (HAADF-STEM) coupled with energy-dispersive X-ray spectroscopy (EDS). X-ray photoelectron spectroscopy (XPS, Veresprobe II/ULVAC-PHI instrument) was performed to demonstrate the chemical bond and electron state of the prepared nanomaterials with an Al Kα monochromatic source. X-ray diffraction (XRD, Ultima IV instrument from Rigaku, utilizing a Cu Kα source, λ = 1.5409 Å) patterns was employed in structure analysis. Fourier transform infrared (FTIR) spectra of the synthesized nanomaterials were obtained using a Shimadzu FTIR spectrometer in the range of 400–4000 cm^{-1}. UV–visible absorption spectral investigation was

performed by a Shimadzu UV-2600i spectrophotometer using a 1 cm path length quartz cuvette with a wavelength range from 200–800 nm. All the electrochemical measurements were conducted using a portable potentiostat/gavanostat (PalmSens4, Netherlands) with integrated SPCEs consisting of carbon working (4 mm diameter), counter electrodes, and an Ag/AgCl reference electrode. Cyclic voltammograms of the bare SPCE, $Ti_3C_2T_x$ MXene/SPCE, SH-$Ti_3C_2T_x$ MXene/SPCE, AuNPs-S-$Ti_3C_2T_x$ MXene/SPCE, and AuNPs-$Ti_3C_2T_x$ MXene were obtained in a phosphate buffer solution (0.1 M PBS, pH 7.0) at a scan rate of 50 mV/s. Electrochemical impedance spectroscopy (EIS) of bare and modified electrodes was performed in 0.1 M KCl containing 2.5 mM $[Fe(CN)_6]^{3-}$ and 2.5 mM $[Fe(CN)_6]^{4-}$ at the formal potential ($E^{o\prime}$) of the redox probe and over a frequency range of 1 MHz to 100 MHz with an amplitude of 10 mV. All the electrochemical results were analyzed at ambient temperature.

2.3. Real Sample Preparation

Milk samples were purchased from a local market in Seoul, South Korea. For the removal of protein, fat, and other ingredients, milk (5 mL) and acetonitrile (5 mL) were added to a 15-mL centrifuge tube. The mixture was vortexed for 10 min and centrifuged at 5000 rpm for 10 min. After the precipitate of a white cream layer was discarded, the supernatant solution was collected, and the above steps were repeated three times. Finally, the resulting supernatant solution was filtered through a Whatman qualitative filter paper to obtain a clear solution, which was then diluted with 0.1 M PBS (pH 7.0) before analysis. Water samples were collected from a Dongguk University lab and the Han River in Seoul, South Korea. These water samples were filtered through the Whatman qualitative filter paper to obtain a clear solution, which was then diluted with 0.1 M PBS (pH 7.0) before analysis.

2.4. Synthesis of $Ti_3C_2T_x$ MXene from Ti_3AlC_2 MAX Phases

$Ti_3C_2T_X$ MXene was synthesized using a previous procedure with slight modifications [1]. Briefly, 2 g of LiF was dissolved in 20 mL of HCl solution (9 M), and the solution was allowed to mix completely at ambient temperature for 30 min with magnetic stirring (350 rpm). Subsequently, 1.25 g of Ti_3AlC_2 was gradually added over the course of 20 mins under magnetic stirring (500 rpm), and the reaction temperature was kept below 5 ºC to prevent the reaction's exothermic nature from leading to overheating. After 1 h, the temperature was raised to 40 ºC, and the reaction was allowed to continue for 48 h with continuous stirring. The etching mechanism can be shown as follows: $Ti_3AlC_2 + 3HF \rightarrow Ti_3C_2T_x + AlF_3 + 3/2H_2$. After etching, the reaction mixture was washed several times with DD water via centrifugation at 5000 rpm until the supernatant became neutral (pH ≥ 6). The concentration of $Ti_3C_2T_x$ MXene was approximately 14–16 mg/mL and then dried at 60 ºC for 12 h. For the delamination process, the resulting $Ti_3C_2T_x$ MXene is then ultrasonicated in 20 mL of DMSO for 1 h and followed by magnetic stirring for 24 to exfoliate the $Ti_3C_2T_x$ MXene multilayer to single-layer MXene nanosheets. The precipitate was washed several times with DD water via centrifugation at 5000 rpm and vacuum dried at 60 ºC for 12 h.

2.5. Synthesis of Thiol-Functionalized $Ti_3C_2T_x$ MXene (SH-$Ti_3C_2T_x$ MXene)

Thiol-functionalized $Ti_3C_2T_x$ MXene (SH-$Ti_3C_2T_x$ MXene) was synthesized using the previously reported procedure with minor modifications [2]. The synthesis of SH-$Ti_3C_2T_x$ MXene was carried out as follows: 0.5 g of vacuum-dried $Ti_3C_2T_x$ MXene was dispersed in 30 mL of anhydrous toluene and ultrasonicated for 30 min, which was followed by the addition of 1 g of MPTMS. Finally, the suspension was refluxed at 80 ºC for 24 h with constant magnetic stirring (500 rpm) for homogeneous suspension so that $Ti_3C_2T_x$ MXene and MPTMS could

react with each other. After 24 h of magnetic stirring, the suspension was separated via centrifugation, washed with DD water (thrice) and ethanol (thrice), and dried at 50 ºC overnight to obtain the SH-$Ti_3C_2T_x$ MXene sheet.

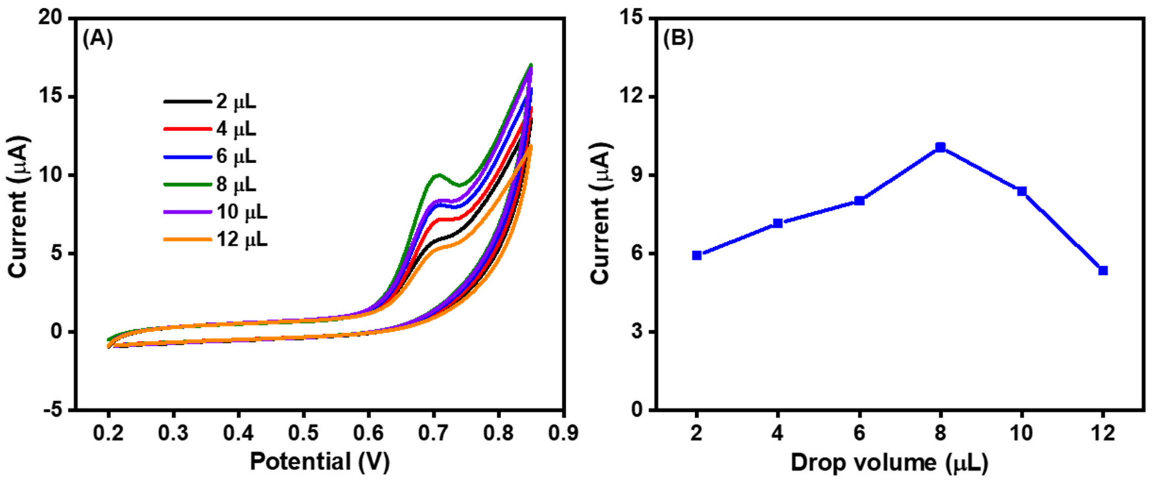

Figure S1. The effect of drop volume optimization. (**A**) Cyclic voltammograms of AuNPs-S-$Ti_3C_2T_x$ MXene sheet in the presence of 150 µM CIPF in 0.1 M PBS at a scan rate of 50 mV/s. (**B**) Corresponding oxidation peak current response vs drop volume.

3. Results and discussion

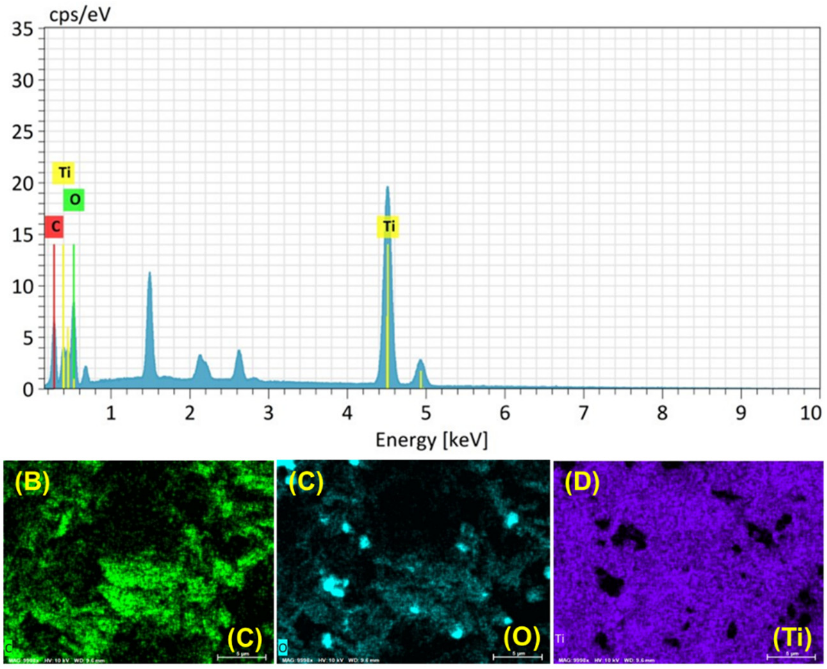

Figure S2. (**A**) EDS analysis and the corresponding elemental mapping of $Ti_3C_2T_x$ MXene: (**B**) carbon, (**C**) oxygen, and (**D**) titanium.

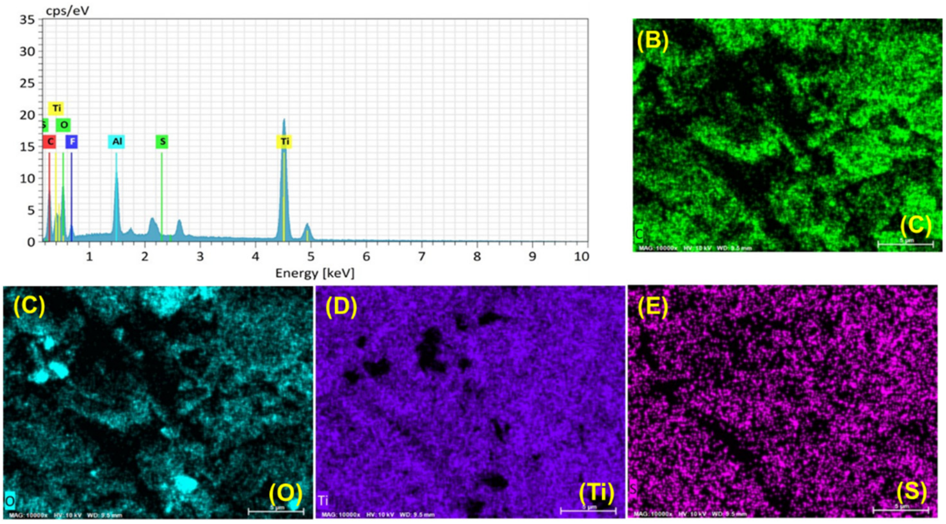

Figure S3. (**A**) EDS analysis and the corresponding elemental mapping of SH-$Ti_3C_2T_x$ MXene: (**B**) carbon, (**C**) oxygen, (**D**) titanium, and (**E**) sulphur.

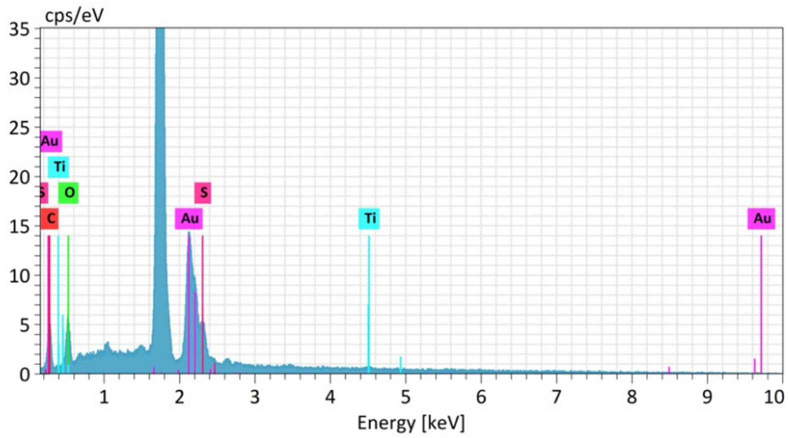

Figure S4. EDS analysis of AuNPs-S-$Ti_3C_2T_x$ MXene.

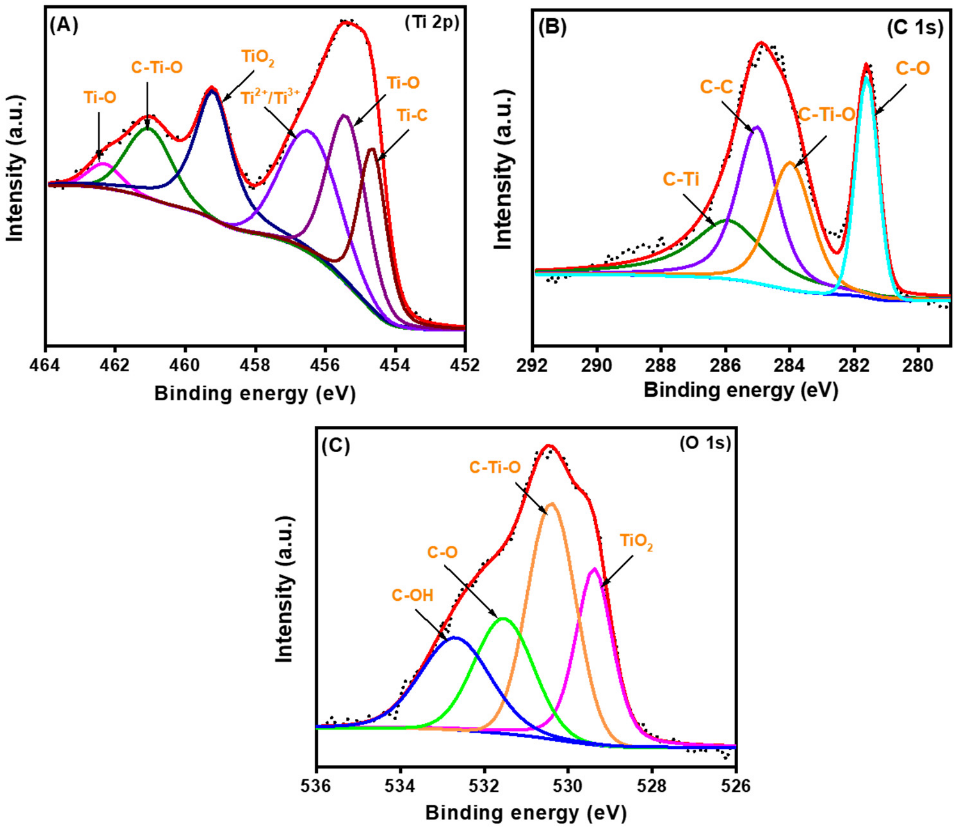

Figure S5. XPS spectra of the (**A**) Ti 2p, (**B**) C 1s, and (**C**) O 1s regions for $Ti_3C_2T_x$ MXene.

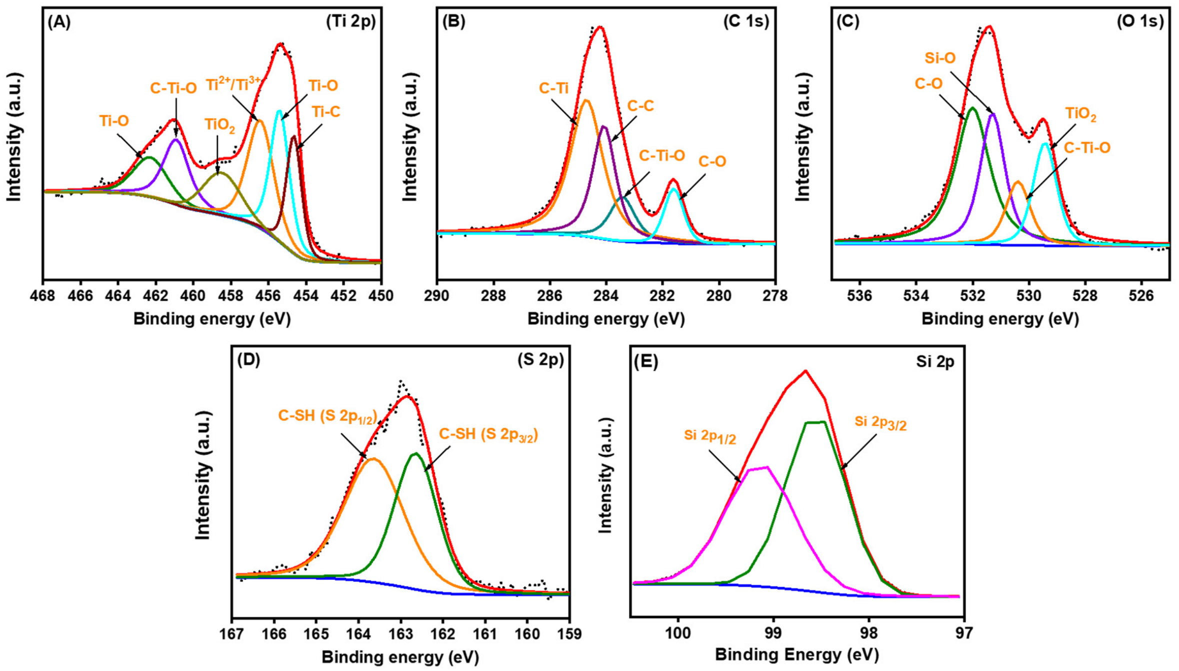

Figure S6. XPS spectra of the (**A**) Ti 2p, (**B**) C 1s, (**C**) O 1s, (**D**) S 2p, and (**E**) Si 2p regions for SH-$Ti_3C_2T_x$ MXene.

3.1. Electroactive Surface Area Calculation for the Bare SPCE and Modified SPCEs

The electroactive surface areas of the bare and modified SPCEs were calculated using the Randles-Ševčík equation for quasi-reversible processes at 25 ºC [3]:

$$I_p = (2.63 \times 10^5)\, n^{3/2} A C D^{1/2} v^{1/2} \text{ ------------------------------ (S1)}$$

where I_p is the peak current (A), n is the number of electrons involved in the redox reaction (unity for $[Fe(CN)_6]^{3-/4-}$), A is the effective surface area of bare and modified SPCEs (cm^2), C is the concentration of the redox probe (mol/cm^3), D is the diffusion coefficient (7.2×10^{-6} cm^2/s for $[Fe(CN)_6]^{3-/4-}$ in 0.1 M KCI) [4], v is the scan rate (V/s). The electroactive surface area were found to be 0.049, 0.068, 0.030, 0.077, and 0.084 cm^2 for the bare SPCE, $Ti_3C_2T_x$ MXene/SPCE, SH-$Ti_3C_2T_x$ MXene/SPCE, AuNPs-$Ti_3C_2T_x$ MXene/SPCE, and AuNPs-S-$Ti_3C_2T_x$ MXene/SPCE, respectively.

3.2. Effect of a Scan Rate

The effect of a scan rate on the voltammetric performance of the fabricated non-enzymatic sensors was studied using CV at various scan rates from 5 to 100 mV/s with 200 µM CIPF in 0.1 M PBS, and the obtained results are portrayed in Figure S7A. As can be seen, the anodic peak current (I_{pa}) progressively increased with the increase in the scan rate. There is a linear relationship with a correlation coefficient (0.996) between the scan rate and the I_{pa} of CIPF, suggesting that the oxidation of CIPF at AuNPs-S-$Ti_3C_2T_x$ MXene/SPCE is an adsorption-controlled process (Figure S7B) [5]. Moreover, when increasing the scan rate from 5 to 100 mV/s, the anodic peak potential (E_{pa}) moves slightly in the positive direction, indicating that the electrochemical oxidation of CIPF at AuNPs-S-$Ti_3C_2T_x$ MXene/SPCE is irreversible. The linear regression equation can be expressed as,

$$I_{pa} = 0.2344 \text{ (mV/s)} + 2.1552 \ (R^2 = 0.996) \text{ --------------------- (S2)}$$

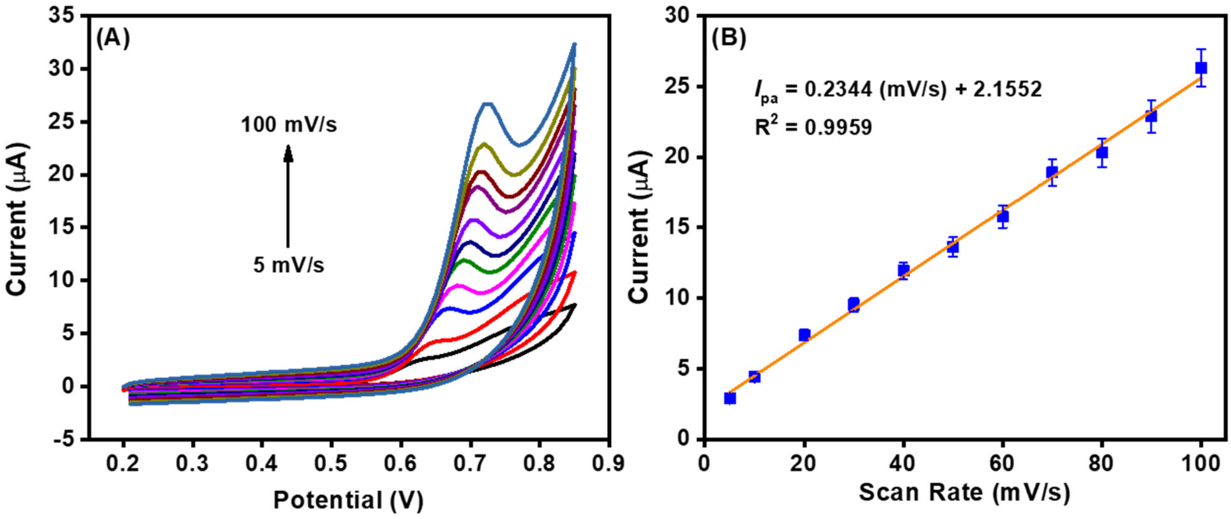

Figure S7. (**A**) Cyclic voltammograms of AuNPs-S-$Ti_3C_2T_x$ MXene/SPCE at various scan rates from 5 to 200 mV/s in 0.1 M PBS (pH 7.0) containing 200 µM CIPF. (**B**) Corresponding linear plot of the oxidation peak currents against the scan rates.

3.3. Effect of pH

The pH of a supporting electrolyte plays an important role in the electrochemical performance of enzymatic and non-enzymatic sensors, and hence, the effect of pH on the fabricated non-enzymatic CIPF sensor was examined from pH 4.0 to 10.0. Figure S8A displays the cyclic voltammograms of the AuNPs-S-$Ti_3C_2T_x$ MXene/SPCE in 0.1 M PBS with varying pH values in the presence of 150 µM CIPF at a scan rate of 50 mV/s. On varying the pH, I_{pa} was found to increase from pH 4.0 to 7.0 and started to decrease beyond pH 7.0. In addition, E_{pa} moves towards a negative direction. The linear relationship between the E_{pa} and pH value was estimated to be 36.4 mV/pH (Figure S8B). It was found that the maximum I_{pa} response was obtained at pH 7.0, and this pH was selected as the optimal pH for further electrocatalytic oxidation of CIPF.

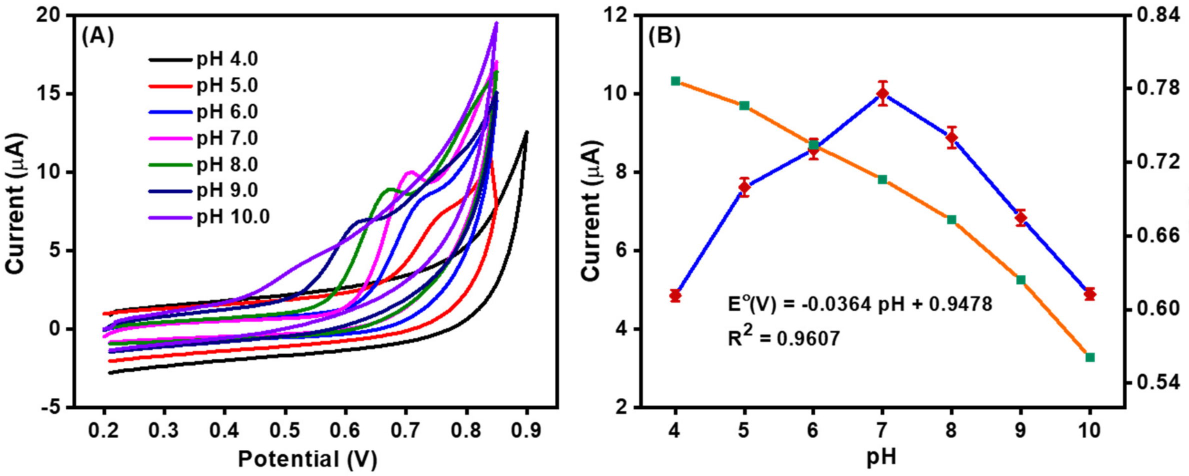

Figure S8. (**A**) Cyclic voltammograms of AuNPs-S-$Ti_3C_2T_x$ MXene/SPCE in 150 µM CIPF at various pH from 4.0 to 10.0 in 0.1 M PBS at a scan rate of 50 mV/s. (**B**) Effect of pH on the anodic peak current (blue line) and oxidation peak potential (orange line) of the AuNPs-S-$Ti_3C_2T_x$ MXene/SPCE.

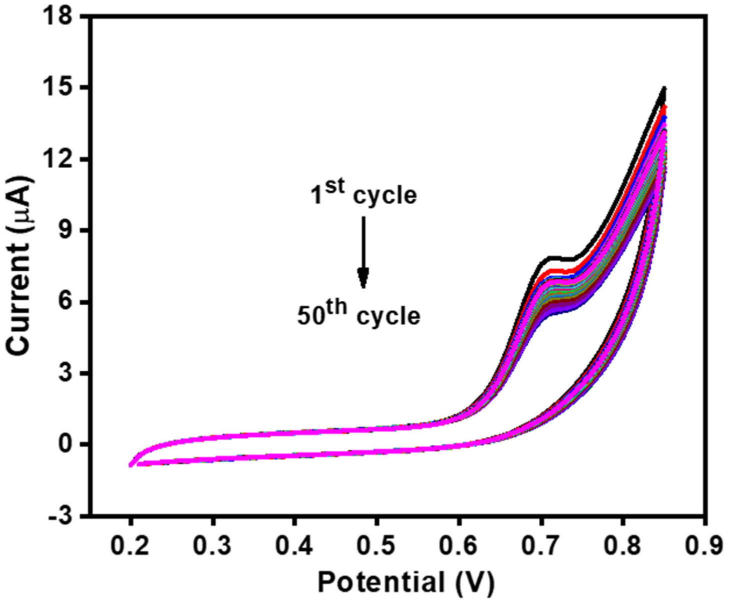

Figure S9. Cyclic voltammograms of AuNPs-S-$Ti_3C_2T_x$ MXene/SPCE for 50 continuous cycles in 100 µM CIPF in 0.1 M PBS (pH 7.0) at a scan rate of 50 mV/s.

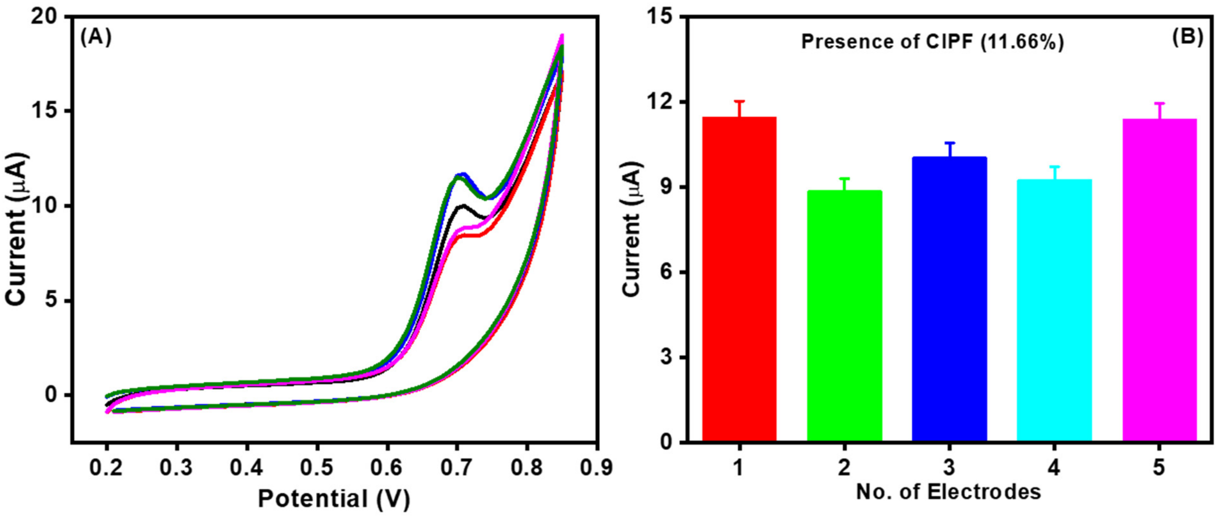

Figure S10. (A) Reproducibility of AuNPs-S-$Ti_3C_2T_x$ MXene/SPCE for the electrocatalytic oxidation of 150 µM CIPF in 0.1 M PBS (pH 7.0) at a scan rate of 50 mV/s. (B) Corresponding columnar diagram of the developed CIPF sensor.

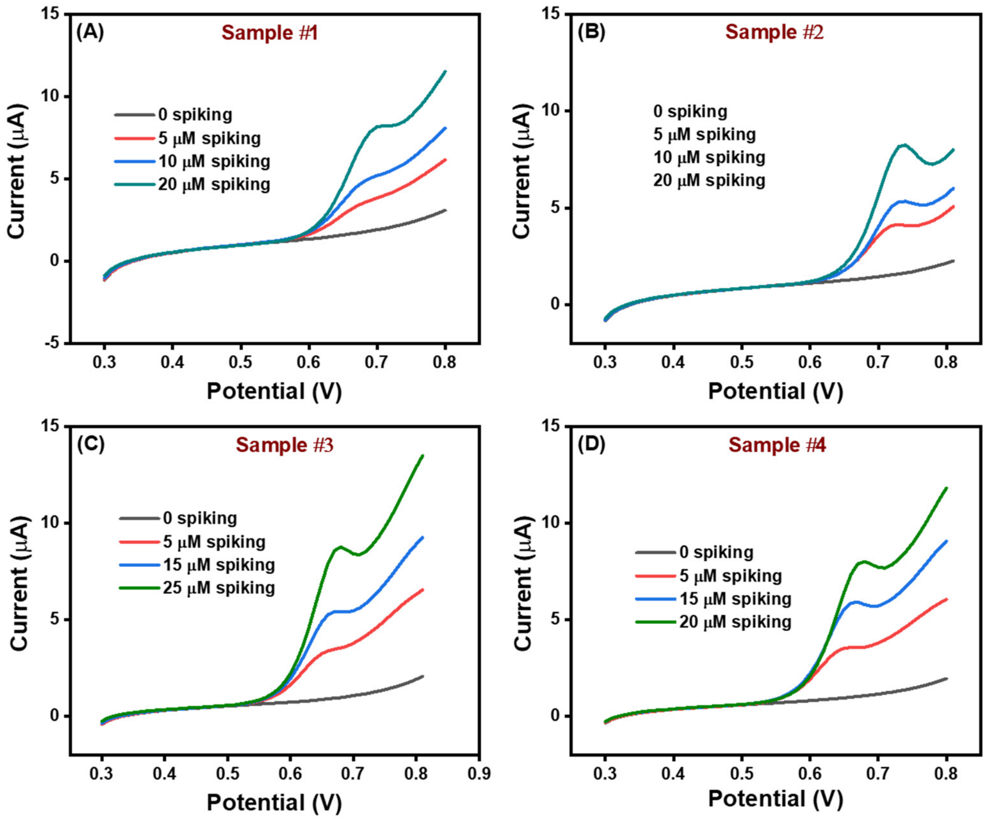

Figure S11. LSV responses of AuNPs-S-$Ti_3C_2T_x$ MXene/SPCE in 0.1 M PBS (pH 7.0) towards the quantification of CIPF in real milk and water samples: (**A**) Milk (removing fat), (**B**) Milk (without removing fat), (**C**) Tap water, and (**D**) River water.

Table S1. Analysis of CIPF spiked in milk and water samples.

Sample	Spiked (μM)	Found (μM)	Recovery (%)	RSD (%)
Milk #1	5.0	4.61	92.2	2.31
	10.0	9.19	91.9	2.50
	20.0	21.5	107.5	2.05
Milk #2	5.0	4.59	91.9	3.26
	10.0	9.38	93.8	2.33
	20.0	21.9	109.6	2.95
Water #3 (tap)	5.0	4.48	89.7	3.53
	15.0	14.41	96.08	2.88
	25.0	27.5	110.3	2.69
Water #4 (river)	5.0	4.77	95.5	2.67
	15.0	14.87	99.1	2.95
	25.0	24.96	99.8	2.45

References

[1] Rong, C; Su, T.; Li, Z.K.; Chu, T.S.; Zhu, M.L.; Yan, Y.B.; Zhang, B.W.; Xuan, F.Z. Elastic Properties and Tensile Strength of 2D $Ti_3C_2T_x$ MXene Monolayers. *Nat. Commun.* **2024,** *15*, 1566.

[2] Bagheri, S.; Chilcott, R.; Luo, S.; Sinitskii, A. Bifunctional Amine- and Thiol-Modified $Ti_3C_2T_x$ MXene for Trace Detection of Heavy Metals. *Langmuir* **2022,** *38*, 12924–12934.

[3] Crapnell, R.D.; Banks, C.E. Perspective: What Constitutes a Quality Paper in Electroanalysis? *Talanta Open* **2021,** *4*, 100065.

[4] Konopka, S.J.; McDuffie, B. Diffusion Coefficients of Ferri-and Ferrocyanide Ions in Aqueous Media, Using Twin-Electrode Thin-Layer Electrochemistry. *Anal. Chem.* **1970,** *42*, 1741–1746.

[5] Zhang, S.; Yu, S.; Wang, X.; Zhang, Y.; Yue, Z.; Li, C.; Ma, Y. An Electrochemical Sensor Based on MnO_2/ZnO Composites for the Detection of Ciprofloxacin in Honey. *Microchem. J.* **2023,** *194*, 109355.
